# Sustainable Hydrogen Photoproduction by Phosphorus-Deprived Marine Green Microalgae *Chlorella* sp.

**DOI:** 10.3390/ijms16022705

**Published:** 2015-01-26

**Authors:** Khorcheska Batyrova, Anastasia Gavrisheva, Elena Ivanova, Jianguo Liu, Anatoly Tsygankov

**Affiliations:** 1 Institute of Basic Biological Problems RAS, Institutskaya 2, Pushchino, Moscow Region 142290, Russia; E-Mails: larisa_nastya@mail.ru (A.G.); cheredova@mail.ru (E.I.); ttt-00@mail.ru (A.T.); 2 Département de Microbiologie, Infectiologie et immunologie, Université de Montréal, CP 6128 Succursale Centre-ville, Montréal, QC H3C 3J7, Canada; 3 Institute of Oceanology, Chinese Academy of Sciences, Chinese Academy of Sciences, 7 Nanhai Road, Qingdao 266071, China; E-Mail: jgliu@ms.qdio.ac.cn

**Keywords:** hydrogen photoproduction, marine green microalgae, phosphorus deprivation, dilution method

## Abstract

Previously it has been shown that green microalga *Chlamydomonas reinhardtii* is capable of prolonged H_2_ photoproduction when deprived of sulfur. In addition to sulfur deprivation (-S), sustained H_2_ photoproduction in *C. reinhardtii* cultures can be achieved under phosphorus-deprived (-P) conditions. Similar to sulfur deprivation, phosphorus deprivation limits O_2_ evolving activity in algal cells and causes other metabolic changes that are favorable for H_2_ photoproduction. Although significant advances in H_2_ photoproduction have recently been realized in fresh water microalgae, relatively few studies have focused on H_2_ production in marine green microalgae. In the present study phosphorus deprivation was applied for hydrogen production in marine green microalgae *Chlorella* sp., where sulfur deprivation is impossible due to a high concentration of sulfates in the sea water. Since resources of fresh water on earth are limited, the possibility of hydrogen production in seawater is more attractive. In order to achieve H_2_ photoproduction in P-deprived marine green microalgae *Chlorella* sp., the dilution approach was applied. Cultures diluted to about 0.5–1.8 mg Chl·L^−1^ in the beginning of P-deprivation were able to establish anaerobiosis, after the initial growth period, where cells utilize intracellular phosphorus, with subsequent transition to H_2_ photoproduction stage. It appears that marine microalgae during P-deprivation passed the same stages of adaptation as fresh water microalgae. The presence of inorganic carbon was essential for starch accumulation and subsequent hydrogen production by microalgae. The H_2_ accumulation was up to 40 mL H_2_ gas per 1iter of the culture, which is comparable to that obtained in P-deprived *C. reinhardtii* culture.

## 1. Introduction

The ability of microalgae to produce hydrogen under the light was discovered more than 70 years ago [1]. Under photoautotrophic conditions the process realizes the true water-splitting reaction.

If one could implement technically this process with H_2_ and O_2_ spatial (or temporal) separation, at low operational cost, in practical scale, and with high rate, the human population could get environmentally friendly and clean energy from a renewable source. However, many fundamental and technical problems should be solved before microalgal hydrogen production comes into practical stage.

Sulfur deprived cultures of microalgae realize temporal separation of oxygen and hydrogen production. Fundamental mechanisms of this process are under active investigations [2,3,4]. However, hydrogen production under S-deprivation is still very expensive. One of the ways to decrease the operational cost is realization of the process in seawater, which is cheaper than fresh water.

In recent years most of research has been focused on developing biological H_2_ production using different fresh water species of *Chlamydomonas*, *Scenedesmus*, and *Chlorella*, with *C. reinhardtii* as a model system [5,6]. Significant advances in H_2_ photoproduction from *C. reinhardtii* have recently been reported [5,7,8], whereas relatively few studies have examined H_2_ production from marine microalgae [9,10,11,12]. Early attempts to produce sustainable hydrogen by marine microalgae under sulfur deprivation were not successful [9]. Marine microalgae *Platymonas subcordiformis* [13] and *Platimonas helgolandica* [14] produced significant quantities of hydrogen under light only after addition of carbonyl cyanide m-chlorophenylhydrazone.

Sustained H_2_ production under sulfur deprivation by marine microalgae is impossible due to high sulfur content in the seawater ~0.028 M·L^−1^ [15], which is even higher than saturating concentrations in TAP medium (0.016 M·L^−1^).

Artificial seawater also cannot produce decent S-deprived conditions due to high sulfur content in salts like NaCl, which is added to the medium at high concentration (~35 g·L^−1^). Extra-pure NaCl (Sigma, product No. 13423) contains sulfates as contaminants (200 mg per 1 kg NaCl), and if used for preparation of artificial seawater will result in none limiting for microalgae concentrations of sulfates (~0.07 mM) [16]. In contrast, concentration of phosphorus in Atlantic and Pacific Ocean varies from 0.07 to 0.2 µM·L^−1^ and depends on the pollution of ocean waters [17].

Taking into account that phosphorus content in seawater is very low, in the present work we developed phosphorus-deprivation for generation of hydrogen production in marine microalgae, *Chlorella* sp*.* We demonstrated that *Chlorella* sp. can produce H_2_ gas under phosphorus deprived conditions by using dilution method that was applied before for fresh water microalgae [18]. Cultures washed of phosphorus and diluted to below 2 mg Chl·L^−1^ in the phosphate-free medium were able to establish anaerobiosis after the growth period and produce H_2_ gas in the similar quantities as phosphorus-deprived fresh water microalgae.

## 2. Results

**Figure 1:**
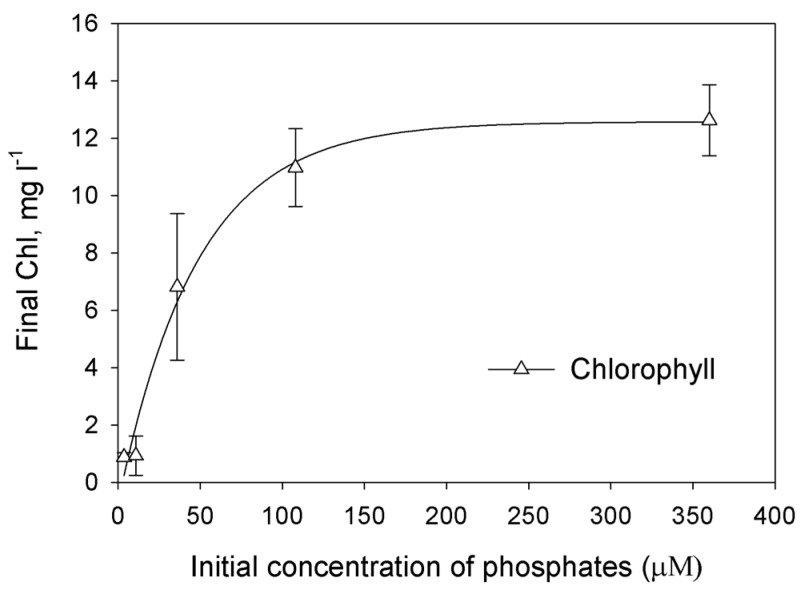
The effect of the initial phosphates concentration on the accumulation of the biomass (measured as the final Chl concentration). Each point represents the average of three independent repetitions. The curve was drawn as nonlinear regression, modified Hyperbola III.

In order to determine limiting initial concentration of phosphates for growth of *Chorella* sp. culture, the dependence of final concentration of biomass (expressed as Chl) on initial phosphates concentration in L1 medium under photoautotrophic conditions was studied (Figure 1). The increase of phosphate-ions concentration from 4 to 12 μM in the medium did not result in increase of the final Chl accumulation. The increase of phosphate-ions concentration from 12 to 36 μM in the medium resulted in significant increase of the final Chl accumulation. One could conclude that 4–36 μM is the region of phosphates concentration when the culture comes to stationary phase due to the lack of the phosphorus. The increase to 108 μM in the medium also resulted in increase of final Chl concentration. Possibly, in the range 36–108 μM phosphorus is also limiting substrate and at the end of growth cultures are P-deprived.

Unfortunately, the photoautotrophic growth of *Chlorella* sp. in artificial seawater was not stable and we could not keep the culture for a long time. That is why other experiments were done with photoheterotrophic cultures using TA-P/SW or TA-P/NaCl media as described in materials and methods section.

**Figure 2:**
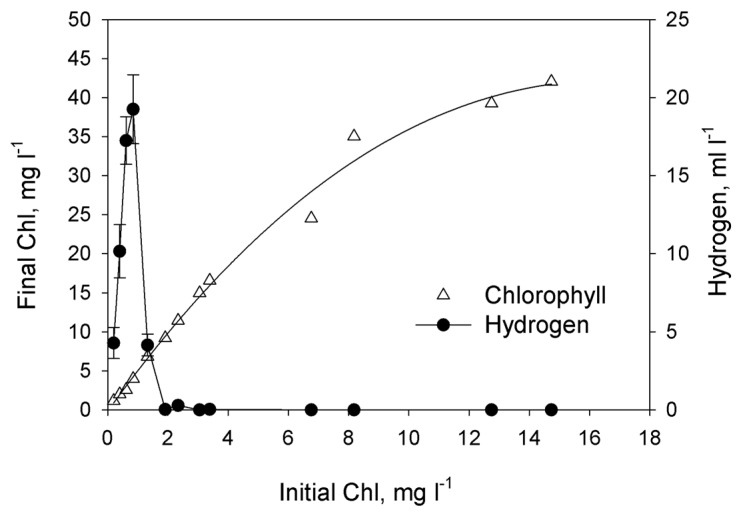
The effect of the initial culture density (measured as the total Chl concentration at the beginning of phosphorus deprivation) on accumulation of the biomass (measured as the final Chl concentration) and the total yield of H_2_ gas produced by the culture. The phosphorus deprivation effect was achieved by using the dilution method. Each point represents the average of three independent repetitions. Curve that represents Chlorophyll was drown as nonlinear regression, modified Hyperbola III. For Hydrogen graph was used simple error bars curve.

In order to achieve sustainable H_2_ production, the dilution method of P-deprivation was applied [18]. This procedure involves phosphorus deprivation by dilution of P-replete cultures into P-free medium. As indicated in Methods section, P-replete cultures were washed in P-free medium and diluted by P-free medium to desired Chl concentrations. In this case initial phosphorus was added together with the cells (and traces of phosphorus in sea and artificial waters). So, the dependence of final Chl concentration on initial ones reflects the influence of initial P concentration on final Chl accumulation. Initial phosphorus content in the medium was regulated by the cells concentration expressed as Chl concentration. As shown in Figure 2, the cell growth (defined as the final Chl concentration in the culture after ~200 h of cultivation) linearly depended on the amount of phosphorus introduced into the culture only when the initial concentration of the total Chl in the culture was below 6.5–8.2 mg Chl·L^−1^. Further increase of initial Chl concentrations resulted in non-linear increase of final Chl concentration. Figure 2 shows that there was a narrow range in initial Chl concentrations leading to the efficient H_2_ photoproduction under phosphorus-deprived conditions. The maximum volume of H_2_ gas (20 ml·L^−1^) was obtained in the culture with the initial Chl content 0.8 mg·L^−1^. Cultures with the initial Chl concentration above 2 mg·L^−1^ did not produce H_2_ gas even if they have evident P-deprivation at the end of growth (as judged from linear dependence of final Chl accumulation on initial ones).

**Figure 3:**
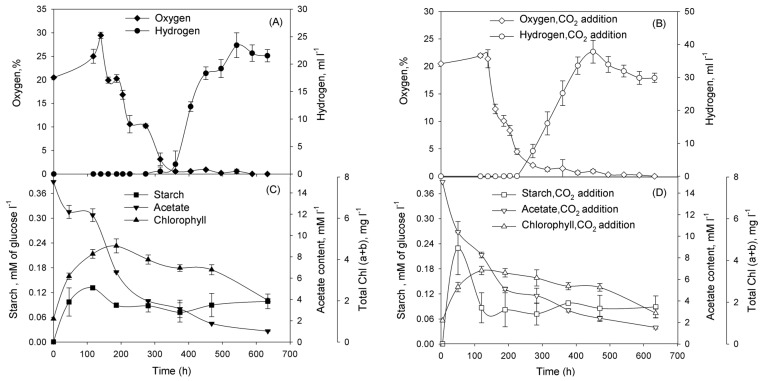
Incubation of *Chlorella* sp. in TA-P/NaCl medium without CO_2_ (**A**,**C**), and with CO_2_ additions (**B**,**D**). At the start of incubation the gas phase was supplemented with CO_2_ to 10% (*v*/*v*). Parameters that were examined: (**A**,**B**) O_2_ concentration in a gas phase of vials (%) and volume of the H_2_ gas produced (mL·L^−1^ of suspension); (**C**,**D**) intracellular starch and acetate in the medium (mM·L^−1^), total (a + b) chlorophyll (mg·L^−1^). Figure represents data of typical experiments from three independent repetitions with the same trend. For all graphs on Figure 3 simple error bars curves were used.

**Figure 4:**
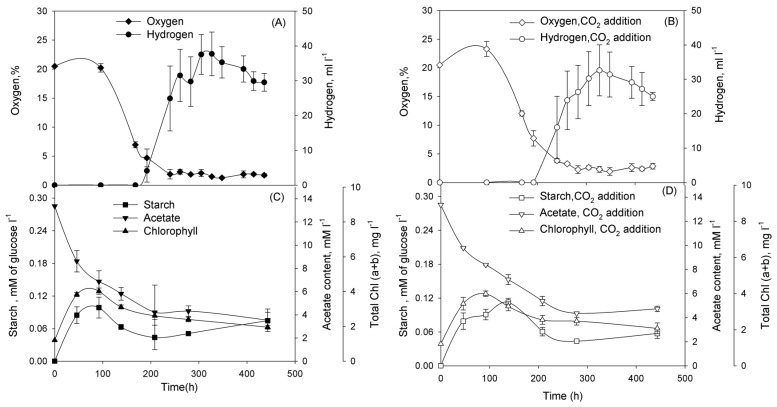
Incubation of *Chlorella* sp. in TA-P/SW medium without CO_2_ (**A**,**C**), and with CO_2_ additions (**B**,**D**). At the start of incubation the gas phase was supplemented with CO_2_ to 10% (*v*/*v*). Parameters that were examined: (**A**,**B**) O_2_ concentration in a gas phase of vials (%) and volume of the H_2_ gas produced (mL·L^−1^ of suspension); (**C**,**D**) intracellular starch and acetate in the medium (mM·L^−1^), total (a + b) chlorophyll (mg·L^−1^). Figure represents data of typical experiments from three independent repetitions with the same trend. For all graphs on Figure 4 simple error bars curves were used.

Subsequently, we performed series of experiments and demonstrated that cultures washed of phosphorus in TA-P/NaCl or TA-P/SW media and diluted to the initial Chl concentration ~1 mg Chl·L^−1^ in either TA-P/NaCl or TA-P/SW media pass through certain physiological stages. In the beginning of incubation cultures grew as usual cultures with oxygen production (Figure 3A,B for TA-P/NaCl and Figure 4A,B for TA-P/SW media). At this stage cultures accumulated starch in both media (Figure 3C,D and Figure 4C,D). Evidently significant reserve of intracellular phosphorus exists allowing the *Chlorella* sp. cultures to grow continuously for several days after removal of phosphates from the medium. Oxygen production stage is followed by the stage of oxygen consumption that was observed in all cultures (Figure 3A,B and Figure 4A,B). At this stage cultures with and without CO_2_ additions in both media started to consume starch (Figure 3C,D and Figure 4C,D). After this stage hydrogen production stage starts in all cultures. This stage is finished and changed by termination stage.

Cultures in TA-P/NaCl medium have longer oxygen production, oxygen consumption, and hydrogen production stages than in TA-P/SW medium independently on CO_2_ presence (Table 1).

**Table 1:** Duration in hours of different stages of *Chlorella* sp. adaptation to P-deprivation.

Culture	Oxygen	Oxygen Consumption Stage **	Hydrogen Production Stage ***
	Production Stage *		
TA-P/NaCl	137.6 ± 6.1	154.7 ± 15.01	214 ± 13.53
TA-P/NaCl + CO_2_	140.6 ± 6.03	124 ± 6.56	221.7 ± 10.41
TA-P/SW	96.3 ± 3.51	93.7 ± 3.21	134.3 ± 6.02
TA-P/SW + CO_2_	98 ± 6.56	102.7 ± 7.505	131 ± 6.56

* Oxygen production stage is defined as the time from the start of experiment to maximum of oxygen in gas phase; ** Oxygen consumption stage is defined as the time from maximum oxygen in gas phase to start of hydrogen production; *** Hydrogen production stage is defined as the time from the start to the end of hydrogen production; Data in the table are average of three independent experiments with standard deviations as defined in Microsoft Office Excel 2007.

Having demonstrated that *Chlorella* sp. cultures are capable for H_2_ photoproduction under phosphorus-deprived conditions, we addressed the question of improving H_2_ production yield in the system. One of the ways to improve the H_2_ production is increasing of starch accumulation during the O_2_-production stage of the phosphorus-deprivation. Therefore, in the next series of experiments, we checked the dependence of starch accumulation and H_2_ production in phosphorus-deprived *Chlorella* sp. on CO_2_ addition. CO_2_ is the additional carbon source for growth and starch accumulation of the culture. The adaptation of cells to phosphorus-deprived conditions is accompanied by the accumulation of starch during the O_2_-production stage. Starch accumulation was increased twofold after carbon dioxide addition during the initial stages of phosphorus-deprivation in TA-P/NaCl medium, H_2_ production was also increased (Figure 3). However, no significant difference was observed, either in starch accumulation or in H_2_ production, in the presence of carbon dioxide during the phosphorus deprivation in TA-P/SW medium (Figure 4). Chemical analysis of seawater revealed the presence of high concentrations of carbonates and hydrocarbonates that served as additional carbon sources for *Chlorella* sp. cultures (Table 2), thus alleviated the effect of carbon dioxide addition. For both cases in TA-P/SW and TA-P/NaCl media starch accumulation was maximal during the O_2_-production stage, but then degradation of starch during the O_2_-consumption stage began (Figure 3C,D and Figure 4C,D). Most of the amount of starch was consumed during the O_2_-consumption stage. The establishment of anaerobiosis under phosphorus deprivation is an energy-dependent process that requires a substrate for respiration. The main substrates for respiration during the period ~100 to 250 h of phosphorus deprivation are acetate and starch (or organic acids produced during starch fermentation), as seen in Figure 3C,D and Figure 4C,D.

**Table 2:** The chemical composition of marine water from Black Sea that was used in the experiments to study phosphorus deprivation in TA-P/SW medium.

Components	Concentration, mg·L^−1^
CO_3_^2−^	11.4 ± 0.8485
НСО_3_^−^	184.22 ± 1.7253
Cl^−^	10530 ± 330.93
SO_4_^2−^	1402.44 ± 15.373
Ca^2+^	258 ± 2.8284
Mg^2+^	675.6 ± 5.0912
K^+^	225.475 ± 6.371
Na^+^	5857.49 ± 187.37
P_2_O_5_	0.005251 ± 0.002
Dry residue	21,055± 63.639

## 3. Discussion

Marine green microalgae offer several advantages for the H_2_ photoproduction. First, the use of marine microalgae will enable us to utilize the unlimited resources of seawater, thereby minimizing the potential use of limited fresh water resources. Secondly, gas solubility is reduced in aquatic saline systems [19], which is potentially advantageous because the levels of soluble O_2_, a potential inhibitor of most FeFe-hydrogenases [20] are diminished in salt water relative to fresh water. Hydrogen is also less soluble in saline systems and therefore more easily removed.

Because little previous research has examined H_2_ production in marine green microalgae, we undertook an effort aimed at optimization of phosphorus deprivation for generation of hydrogen production in marine green microalgae *Chlorella* sp. We first checked how exclusion of phosphorus from the medium affects H_2_ photoproduction in marine green microalga. The most direct and evident way to prove that microalgae experience phosphorus starvation is to find the region of initial P where microalgal final concentration depends on initial P. The dependence can be linear and non-linear. In case of linear dependence the culture is limited by phosphorous. Non-linear dependence occurs when the culture experience double limitation as in the case of ammonium-limited cultures of purple non-sulfur bacterium *Rhodobacter capsultus* which have region of double limitation by ammonium and by light [21]. Using photoautotrophic cultures we were able to produce direct experiments changing initial phosphorus concentration (Figure 1). In the range from 4 to 12 μM of added P we did not observe significant changes of final Chl concentration. Possibly, in this case intracellular P has more impact to the growth of the culture. At higher concentrations cultures showed evident dependence of final Chl on added P up to 36 or even 108 μM proving that in this region we have P-limited cultures, which were deprived of P at the end of growth. Unfortunately, photoautotrophic cultures did not grow stably in artificial seawater. Taking into account that photoautotrophic hydrogen production usually needs particular light conditions [22], which should be optimized for particular culture; in the rest of our study we used photoheterotrophic and photomixotrophic cultures of *Chlorella* sp. for hydrogen production experiments. For these cultures we applied a dilution approach that was previously used for obtaining H_2_ production in sulfur-deprived [23] and phosphorus-deprived *C. reinhardtii* cultures [18]. In this approach, cells utilize limited nutrients accumulated inside cells during the growth phase and after that become nutrient-limited and, very soon after, nutrient-deprived. We demonstrated that final microalgal concentration depends linearly on initial cells concentration up to ~8 mg Chl·L^−1^ (Figure 2). Since the medium was without added P, we assume that the growth of the culture was restricted by P availability. Surprisingly, hydrogen production was observed in very narrow range of initial cells concentration with sharp maximum near 0.8 mg·Chl·L^−1^.The decrease in H_2_ production in the region lower than 0.8 mg·Chl·L^−1^ can be attributed to the low final concentration of the culture. The absence of hydrogen production in the range 2–8 mg·Chl·L^−1^ is more complicated for the explanation. This is the range of phosphorous deprivation at the end of growth. One could suggest that at higher initial concentrations of cells cultures growth to higher final Chl and acetate was consumed before the anaerobiosis establishment. However, additional research is necessary to clarify this suggestion. Nevertheless, our experiments clearly showed that phosphorus-deprived cultures of marine microalga *Chlorella* sp. are able to produce hydrogen.

Our data show that the phosphorus-deprived culture passes through following stages: stage of the growth, starch accumulation, and oxygen production; stage of oxygen consumption; stage of hydrogen production; and termination stage. Transition of cultures from oxygen production to oxygen consumption stage can be clearly differentiated by the point when the rate of photosynthesis is equal to the rate of respiration. Future research should clarify what is the difference in microalgal adaptation to oxygen production and oxygen consumption stages but these stages are different in physiological appearance. That is why these stages should be separated from each other rather than joint in the single, oxygen stage. The set of stages described above differs from phosphorus-deprived fresh water cultures of microalgae [18] by the visible absence of anaerobic stage. However, taking into account that we measured oxygen in gas phase, culture could pass through anaerobic stage when oxygen still present in the gas phase but is equal to zero in the liquid. This suggestion is supported by the fact that in some cases hydrogen appeared when oxygen concentration in gas phase is rather high (see, for example, Figure 4A). Hydrogen production by microalgae is the good indication of anaerobiosis of the culture [2]. Thus, taking into account that anaerobic stage has duration from hours [24] to days [22] and that for the hydrogenase induction is important [25] we could conclude that marine microalgae also pass through anaerobic stage. Summarizing our considerations, it is possible to claim that marine microalgae *Chlorella* sp. passes the same stages as fresh water microalgae during phosphorus deprivation.

The experiments with CO_2_ addition demonstrated that H_2_ production can be improved by increasing of starch accumulation. The interplay between starch accumulation during the phosphorus-deprivation in TA-P/NaCl and the amount of H_2_ gas produced (see Figure 3) suggests that it is possible to increase hydrogen production further. For example, manipulating the culture conditions at different steps of the process it was possible to increase hydrogen production by photoautotrophic microalgae [22] using cultures with particular pre-history and separating light regimes during oxygen production and hydrogen production stages. Also, taking into account the durability of oxygen consumption stage and quantity of consumed starch, it is possible to suggest that artificial exclusion of oxygen at this stage (for example, bubbling the culture with argon) will accelerate this stage and keep the starch for following hydrogen production. Clearly, more work is needed to accurately define the metabolic pathways and functional regulations involved in the H_2_ photoproduction process under phosphorus-deprived conditions in marine green microalgae *Chlorella* sp., however, the response of *Chlorella* sp. to phosphorus deprivation demonstrates significant similarities with fresh water *C. reinhardtii*: cultures passed through the same physiological stages, including oxygen production with accumulation of starch, oxygen consumption, evidently due to inactivation of PSII, establishment of anaerobiosis in cultures, H_2_ photoproduction and termination.

In conclusion, we have demonstrated for the first time the sustainable H_2_ photoproduction in marine green microalgae *Chlorella* sp. under phosphorus-deprived conditions. In the presence of carbon dioxide, the system accumulated significant amounts of starch during the initial, photosynthetic stage of phosphorus deprivation and produced more hydrogen.

## 4. Methods

### 4.1. Cell Growth

The unicellular marine green algae *Chlorella* sp. IOAC707S was isolated from water samples collected at the Yellow Sea near the coast of Quingdao, China. Two types of media were used to create high salinity conditions for culturing *Chlorella* sp.: Tris-Acetate-Phosphate medium [26] with addition of 30 g·L^−1^ of pure NaCl (TAP/NaCl), and TAP medium where distilled water was substituted with sea water from the Black Sea (TAP/SW). Sea water samples were taken in 1 km of the coast Gelendzhik, Russia at the depth of 50 m. Prior using, sea water samples were filtered using 0.45 µm filters (Nalgene), both types of medium contained 1 mL·L^−1^ of vitamin mix: Cyanocobalamin 0.0005 g·L^−1^, Thiamine HCl 0.1 g·L^−1^, Biotin 0.0005 g·L^−1^. *Chlorella* sp. IOAC707S was grown photomixotrophically on either (TAP/NaCl) or (TAP/SW) media in flat glass bottles at 28 °C, pH 7.2. Algal cultures were bubbled continuously with 2% CO_2_ in air using autoclavable membrane filters with a 0.2 µm pore size (Pall, Ann Arbor, MI, USA). During the growth, the algae were illuminated from two sides with cool white fluorescence lamps providing of about 25 µmol·m^−2^·s^−1^ PAR on the bottle surface (measured with a quantum radiometer photometer Model LI-250, LI-COR, Lincoln, NE, USA). Cells were harvested and used in experiments when the concentration of Chl was 25 mg per liter.

Experiments for studying the dependence of final Chl accumulation on initial concentration of phosphates were performed under photoautotrophic conditions. Cultures *Chlorella* IOAC707S were cultivated during 12 days under continuous illumination in L1 medium, prepared in artificial seawater [15]. Conditions in terms of pH of medium, cultivation temperature, light intensities, purging with air + CO_2_ gas mixture, were the same as indicated above. Medium with predefined concentration of phosphorus was inoculated (1% *v*/*v*) by the culture grown in the same medium. Biomass accumulation was estimated as Chl concentration.

### 4.2. H_2_ Photoproduction in Phosphorus-Deprived Marine Green Microalgae Chlorella sp.

Cultures grown as above on the TAP/NaCl or TAP/SW were washed once in either phosphorus-free TA-P/NaCl or TA-P/SW media (depending on the growth medium) by centrifugation and inoculated into the same medium at different Chl concentrations: ~0.15–20 mg·L^−1^ (see [23] for details). Hydrogen photoproduction in phosphorus-deprived *Chlorella* sp. IOAC707S culture was studied in 45 mL sealed vials (Belco Glass Inc., Vineland, NJ, USA) filled with 30 mL of cells suspension in TA-P/NaCl or TA-P/SW media and sealed with gas-tight, butyl-rubber stoppers. At the beginning of the experiment gas phase of all vials contained air.

When the effect of carbon dioxide addition was investigated, at the beginning of the experiment in the gas phase (initially air) of vials carbon dioxide was injected to 10%, and the pressure in vials was equilibrated with atmosphere. The vials were then placed on an orbital shaker (100 rotations per min) and illuminated from the top with cool-white fluorescent light (~45 µE·m^−2^·s^−1^ PAR) at 25 °C. The gas phase in the vials was analyzed by gas chromatography as described earlier [23].

### 4.3. Other Analytical Procedures

Total chlorophyll (Chl a and Chl b) is abbreviated through the text as Chl. It was determined spectrophotometrically by extraction in 100% methanol and calculated as described earlier [18]. *Chlorella* sp. IOAC707S cells were washed with two volumes of MilliQ water to remove salt prior to pigment extraction. The samples for starch, Chl and acetate contents were taken anaerobicaly directly from the vials with a sterile syringe and pelleted by centrifugation at 13,000 rpm (MiniSpin, Eppendorf, NY, USA) for 3 min. The pellets and supernatants were separated and stored frozen at −20 °C until all samples were ready for processing. The amount of starch accumulated inside the cells was determined in the pellet according to the method developed by Gfeller and Gibbs [18]. The levels of acetate in supernatants were determined by a GC as described earlier [18]. The chemical analysis of Black Sea water samples was processed by bio-chemical laboratory of Institute of Physicochemical and Biological Problems of Soil Science RAS (IPBPSS RAS) Pushchino, Russia. All graphs that presented in the manuscript were made in SigmaPlot 11.

## 5. Conclusions

The presented work has demonstrated for the first time the sustainable H_2_ photoproduction in marine green microalgae *Chlorella* sp. under phosphorus-deprived conditions. In the presence of carbon dioxide, the system accumulated significant amounts of starch during the initial, photosynthetic stage of phosphorus deprivation and produced more hydrogen.
